# Syndecan-1-Induced ECM Fiber Alignment Requires Integrin αvβ3 and Syndecan-1 Ectodomain and Heparan Sulfate Chains

**DOI:** 10.1371/journal.pone.0150132

**Published:** 2016-02-24

**Authors:** Ning Yang, Andreas Friedl

**Affiliations:** 1 Department of Pathology and Laboratory Medicine, University of Wisconsin-Madison, Madison, Wisconsin, United States of America; 2 Pathology and Laboratory Medicine Service, William S. Middleton Memorial Veterans Hospital, Department of Veterans Affairs Medical Center, Madison, Wisconsin, United States of America; 3 UW Carbone Cancer Center, Madison, Wisconsin, United States of America; Fox Chase Cancer Center, UNITED STATES

## Abstract

Expression of the cell surface proteoglycan syndecan-1 (Sdc1) is frequently induced in stromal fibroblasts of invasive breast carcinomas. We have recently identified a correlation between stromal Sdc1 expression and extracellular matrix (ECM) fiber alignment, both *in vitro* and *in vivo*. ECMs derived from Sdc1-positive human mammary fibroblasts (HMF) showed an aligned fiber architecture, which contrasted markedly with the more random fiber arrangement in the ECM produced by Sdc1-negative HMFs. We further demonstrated that aligned fiber architecture promotes the directional migration and invasion of breast carcinoma cells. To decipher the molecular mechanisms governing the formation of an aligned, invasion-permissive ECM, a series of Sdc1 mutants was introduced into HMF. We found that both the ectodomain and heparan sulfate chains of Sdc1 were required for full activity of Sdc1 in regulating ECM alignment, while transmembrane and cytoplasmic domains were dispensable. Sdc1 regulates the activities of several integrins via its ectodomain. Integrins are key players in the assembly of fibronectin-rich ECM. In addition, integrins are capable of regulating cell morphology and cell shape and orientation may affect ECM architecture. Therefore, we investigated the role of integrins in Sdc1-mediated ECM fiber alignment. Sdc1-overexpressing HMF gained an enhanced spindle-shaped morphology when cultured in an overconfluent state under conditions permissive for ECM production, which was partially reversed by siRNA-mediated silencing of β3 integrin expression. Moreover, suppression of αvβ3 integrin activity by a function-blocking antibody or β3 knockdown largely abolished the aligned ECM fiber architecture and consequently the invasion-permissive properties of the ECM induced by Sdc1. The results suggest that Sdc1 may modulate fibronectin fibrillogenesis and/or alter cell morphology during ECM production through αvβ3 integrin, thereby mediating ECM fiber alignment. Understanding the mechanisms governing ECM organization may lead to the development of novel stroma-targeted therapy for breast cancer, aiming at converting an invasion-permissive to an invasion-restrictive microenvironment.

## Introduction

During carcinogenesis, the malignant epithelium and the stroma co-evolve and alterations in cancer stroma contribute to the malignant phenotype. A major acellular component of the tumor stroma is the extracellular matrix (ECM), a three-dimensional (3D) network of proteins, glycoproteins, proteoglycans and polysaccharides. In addition to serving as a structural scaffold, the ECM regulates a wide variety of key cellular events including adhesion, migration, proliferation, differentiation and survival (for reviews see [1,2]). Under normal physiological conditions, the ECM dynamics (production, remodeling and degradation) are precisely regulated to ensure normal organ development and tissue homeostasis. In many cancer types, abnormal deposition of ECM components, resulting in alterations in the biochemical properties of the ECM that promote cancer progression, has been observed [3,4]. Recent evidence indicates that the physical properties of the ECM such as rigidity, topography and architecture are also altered in cancers and that these changes may play key roles in facilitating cancer invasion and progression [5–13]. The fibroblast is the major contributor to the synthesis and remodeling of the ECM and is a prevalent cell type within the tumor stroma. To date, the molecular mechanisms by which the stromal fibroblasts modulate the topography and fine structure of the ECM remain largely unknown.

Sdc1 belongs to a four-member family of cell surface heparan sulfate proteoglycans (HSPGs) [14]. Sdc1 is composed of a transmembrane core protein and covalently coupled heparan sulfate (HS) and chondroitin sulfate (CS) glycosaminoglycan (GAG) chains. Via its HS chains, Sdc1 interacts with a wide range of molecules, functions as a co-receptor to a variety of growth factors and interacts with a number of ECM components. Via its core protein, Sdc1 regulates the activities of integrins and associates with the actin cytoskeleton (for reviews see [15,16]). Consequently, Sdc1 regulates cell proliferation, adhesion, migration, cytoskeletal organization and mediates cell-cell and cell-ECM interactions. In adult organisms, Sdc1 is expressed predominantly by epithelial cells and mature plasma cells, although it is also found in mesenchymal cells and lymphoid cells during development [17–19]. Dysregulated expression of Sdc1 has been reported in many types of cancers and been linked to cancer cell proliferation, invasion, metastasis, angiogenesis, and predicts poor prognosis [20–24].

Our previous work indicated that stromal Sdc1 functions as a critical regulator of ECM architecture [25]. Sdc1 is frequently aberrantly expressed by stromal fibroblasts in invasive breast carcinomas [26,27]. *In vivo* and *in vitro*, stromal syndecan-1 expression correlates with a dramatically altered ECM fiber architecture. ECMs produced by Sdc1-expressing human mammary fibroblasts show an organized, aligned fiber architecture, which contrasts markedly with the haphazard fiber arrangement observed in the ECM produced by Sdc1-negative fibroblasts. Moreover, the structurally abnormal ECM derived from Sdc1-positive fibroblasts promotes the directional migration and invasion of the breast carcinoma cells [25].

In the present study, we identify the molecular regions within Sdc1 that are essential for its activity as an ECM organizer. We find that both the ectodomain and HS chains of Sdc1 are required for the production of aligned ECM fiber architecture. In addition, we show that activity of the integrin αvβ3 is not only involved in Sdc1-induced spindle-shaped morphology of fibroblasts but also required for Sdc1-dependent ECM fiber alignment. We propose that Sdc1 may modulate fibronectin fibrillogenesis through both direct and indirect mechanisms and/or regulate cell morphology, thereby mediating ECM fiber alignment.

## Results

### The Sdc1 ectodomain and HS chains are required for ECM alignment

**Fig 1:**
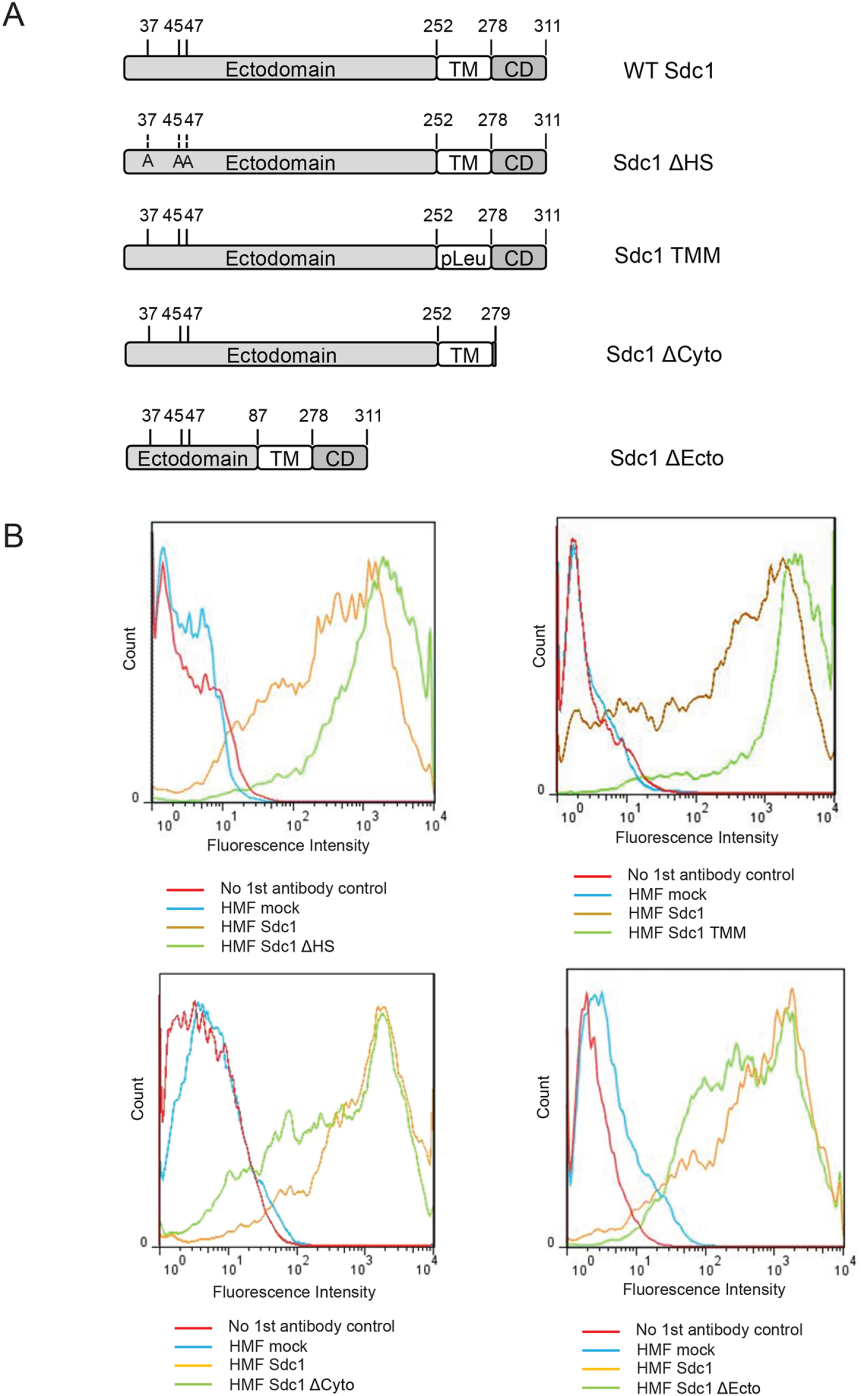
Generation of stable HMF cell lines expressing mutant forms of Sdc1. A) Schematic representation of Sdc1 mutant constructs introduced into HMF cells. B) Flow cytometry analysis of exogenous Sdc1 in stably transfected HMF reveals that wildtype Sdc1 and Sdc1 mutants are expressed at comparable levels.

We have shown previously that Sdc1 functions as a critical regulator during ECM production and that Sdc1-expressing human mammary fibroblasts which are frequently found in the stroma of human breast carcinomas give rise to an aligned, invasion-permissive ECM [25]. To decipher the molecular mechanisms by which Sdc1 modulates the ECM architecture, we first set out to identify the functional domains of Sdc1 that are required for its activity as an ECM organizer. The Sdc1 molecule consists of a core protein and covalently attached GAG chains, three of which are HS chains near the N-terminus. The core protein of Sdc1 can be divided into 3 functional domains: the extracellular domain (ectodomain), the transmembrane domain and the cytoplasmic domain. HMF were stably transfected with a series of Sdc1 mutants and the ECMs produced by these cells were compared to ECMs produced by mock-transfected (negative control) or wild-type Sdc1-transfected HMF. These Sdc1 mutants (schematically shown in Fig 1A) include Sdc1-ΔHS (a HS-deficient mutant in which the three serine residues S37, S45, and S47 required for HS chain attachment are mutated to alanines) [28], Sdc1-TMM (a transmembrane domain substitution mutant in which all the amino acid residues of the transmembrane domain are replaced with leucine residues) [29], Sdc1-ΔCyto (a cytoplasmic domain deletion mutant in which all but the first amino acid residue of the cytoplasmic domain are deleted) [30], and Sdc1-ΔEcto (an ectodomain truncation mutant in which all but the 87 N-terminal amino acids of the extracellular domain are deleted while the three HS chain attachment sites remain unaffected) [31,32]. Populations of HMF expressing these Sdc1 mutant proteins at levels similar to wild-type Sdc1 were enriched by fluorescence-activated cell sorting (FACS) and the expression levels were confirmed by flow cytometry (Fig 1B).

**Fig 2:**
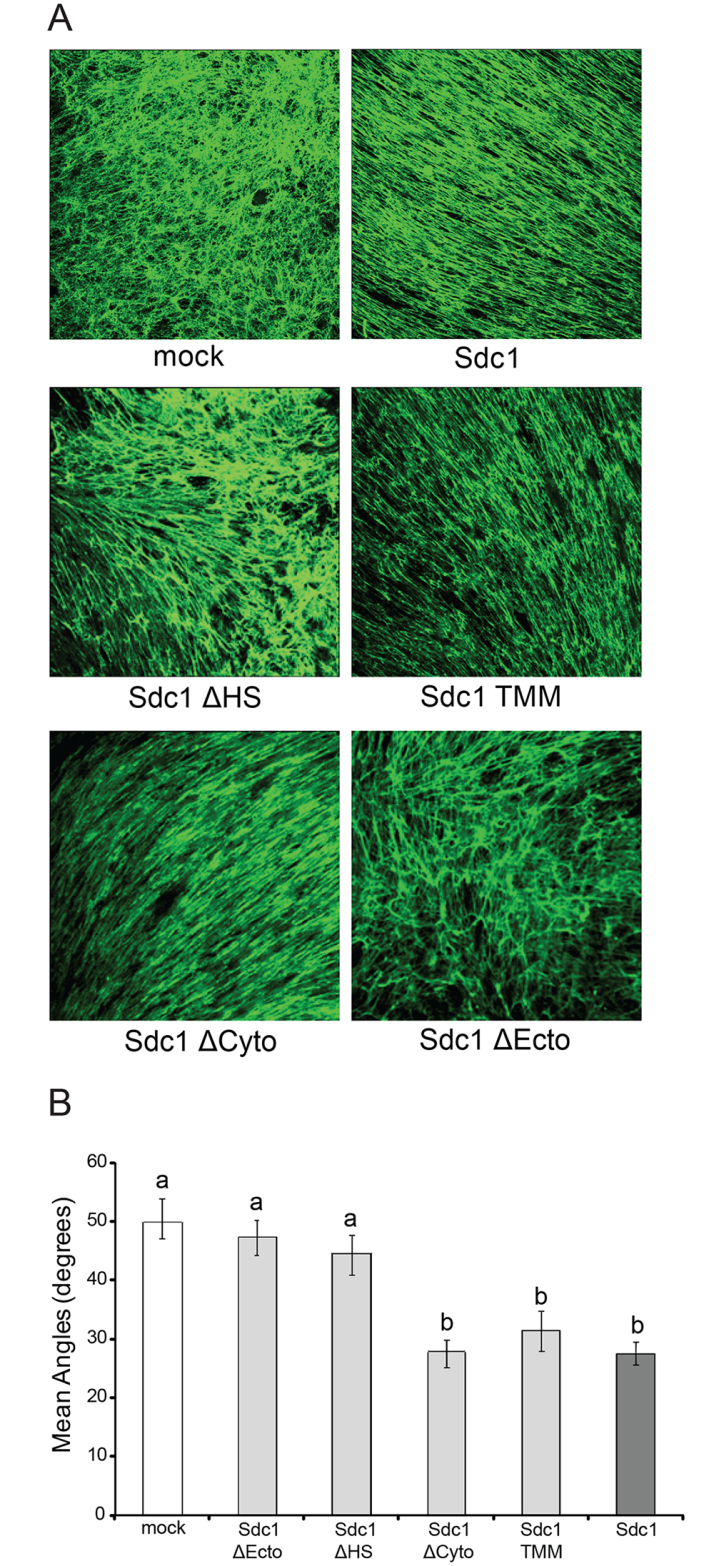
Sdc1 ectodomain and HS chains are required for full activity in regulating ECM fiber alignment. A) Representative confocal microscopy images of cell-free 3D ECMs derived from mock transfected HMF cells (mock), HMF cells expressing wildtype (Sdc1) or mutant Sdc1 (Sdc1-ΔHS, Sdc1-TMM, Sdc1-ΔCyto and Sdc1-ΔEcto). Fibronectin (FN) (green signal) in the ECMs was antibody-labeled and detected by confocal microscopy (Original magnification: 200x). B) Mean fiber-to-fiber angles of HMF-derived ECMs as indicated. ECM fiber angles were measured as described in Materials and Methods. More than 200 fiber-to-fiber angles were quantified for each Sdc1 mutant. Letters above the columns indicate the results of statistical comparisons by ANOVA. Columns sharing the same letter are not significantly different; columns labeled with different letters are significantly different (p<0.001).

The architecture of the 3D ECMs produced by these fibroblasts was examined by confocal microscopy of fibronectin (FN)-immunolabeled ECM preparations (Fig 2A). The expression of Sdc1 greatly affected the topography of the fibroblast-derived ECMs. Consistent with our previous findings, HMF expressing wild-type Sdc1 generated an ECM (ECM-Sdc1) with an organized, parallel FN fiber orientation, which contrasted dramatically with the haphazard fiber arrangement in the ECM produced by the Sdc1-negative HMF (ECM-mock). The transmembrane and cytoplasmic domains of Sdc1 were dispensable for Sdc1-mediated ECM alignment, since fibers in the ECMs derived from HMF that expressed Sdc1-ΔCyto and Sdc1-TMM (ECM-Sdc1-ΔCyto and ECM-Sdc1-TMM) were organized in parallel patterns closely resembling those in ECM-Sdc1. In contrast, HMF that express Sdc1-ΔEcto or Sdc1-ΔHS generated ECMs (ECM-Sdc1-ΔEcto and ECM-Sdc1-ΔHS) with random fiber arrangement, indicating that the Sdc1 HS chain and the part of the ectodomain located between the HS attachment sites and transmembrane domain are required for its full activity as an ECM organizer. Quantitative analysis confirmed that the mean fiber-to-fiber angles in ECM-Sdc1-ΔEcto and ECM-Sdc1-ΔHS are significantly larger than those in ECM-Sdc1 (p<0.001 for both ECM-Sdc1-ΔEcto and ECM-Sdc1-ΔHS) and statistically indistinguishable from ECM-mock. The mean fiber-to-fiber angles in ECM-Sdc1-ΔCyto and ECM-Sdc1-TMM are not significantly different from those in ECM-Sdc1 (Fig 2B).

**Fig 3:**
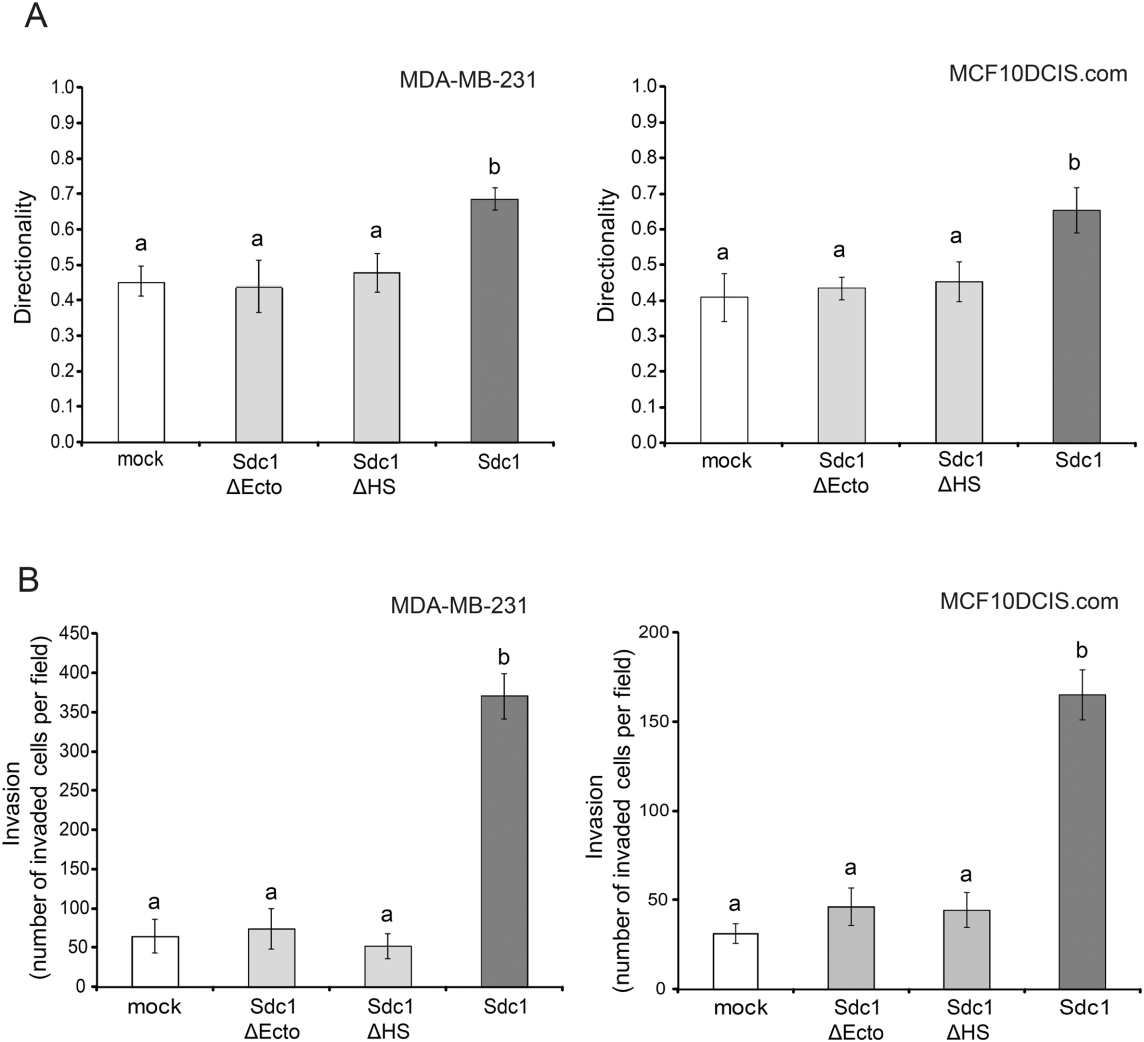
Sdc1 ectodomain and HS chains are required for producing an ECM that promotes directional migration and invasion. A) Time-lapse migration study of MDA-MB-231 and MCF10DCIS.com cells in 3D ECMs derived from HMF-mock cells, HMF-Sdc1 cells, HMF cells expressing the HS-deficient Sdc1 (Sdc1-ΔHS) and HMF cells expressing the ectodomain-truncated Sdc1 (Sdc1-ΔEcto). Cell movements were recorded every 30 minutes for a period of 5–6 hours. The directional persistence of cell migration was determined as the ratio of the migration distance (net distance in a direct line from start to end point) to the total distance traveled. Columns labeled with different letters are significantly different (p<0.001). B) Invasion of MDA-MB-231 and MCF10DCIS.com cells through the indicated HMF-derived ECMs deposited in the inserts of Matrigel invasion chambers. Invasion is reported as the number of invading cells per field. Columns labeled with different letters are significantly different (p<0.001).

We and others had previously determined that mammary fibroblast-derived ECMs with an aligned fiber architecture promote the directionally persistent migration of breast carcinoma cells [11,25]. Therefore, we analyzed the migration of 2 breast carcinoma cell lines, MDA-MB-231, highly invasive, triple-negative cells and MCF10DCIS.com, a model of ductal carcinoma in situ, within the different ECMs using a time-lapse motility assay (Fig 3A). Indeed, in cell-free ECM-Sdc1-ΔHS and ECM-Sdc1-ΔEcto, the majority of carcinoma cells migrated in apparently random and changing directions, similar to the movement observed in ECM-mock. The directionality of migration (which was calculated as the ratio of net distance between starting point and end point to the total distance traveled) of MDA-MB-231 and MCF10DCIS.com cells in these three ECMs had values of less than 0.5. In contrast, both breast carcinoma cell lines migrated with significantly more persistent directionality in ECM-Sdc1. We also examined the invasion of the 2 breast carcinoma cell lines through the different cell-derived ECMs with a transwell chamber-based assay. Compared to ECM-mock, a significantly greater number of cancer cells invaded through ECM-Sdc1 (Fig 3B). However, ECM-Sdc1-ΔEcto and ECM-Sdc1-ΔHS were less invasion-permissive than ECM-Sdc1 and indistinguishable from ECM-mock. These results further confirm that the HS chains and the ectodomain of Sdc1 are required for the production of an ECM that promotes persistent directional migration and invasion of carcinoma cells and thus would be expected to stimulate tumor progression.

### Integrin αvβ3 activity is required for Sdc1-mediated ECM fiber alignment

It has been shown that Sdc1 regulates the activities of several integrins, including αvβ3, αvβ5 and α2β1, via its ectodomain [32–36]. Since certain integrins play crucial roles in the initiation of FN fibrillogenesis [37–39]—a critical event in ECM assembly—we examined the possibility that Sdc1 regulates ECM fiber alignment by activating integrin ECM adhesion receptors.

**Fig 4:**
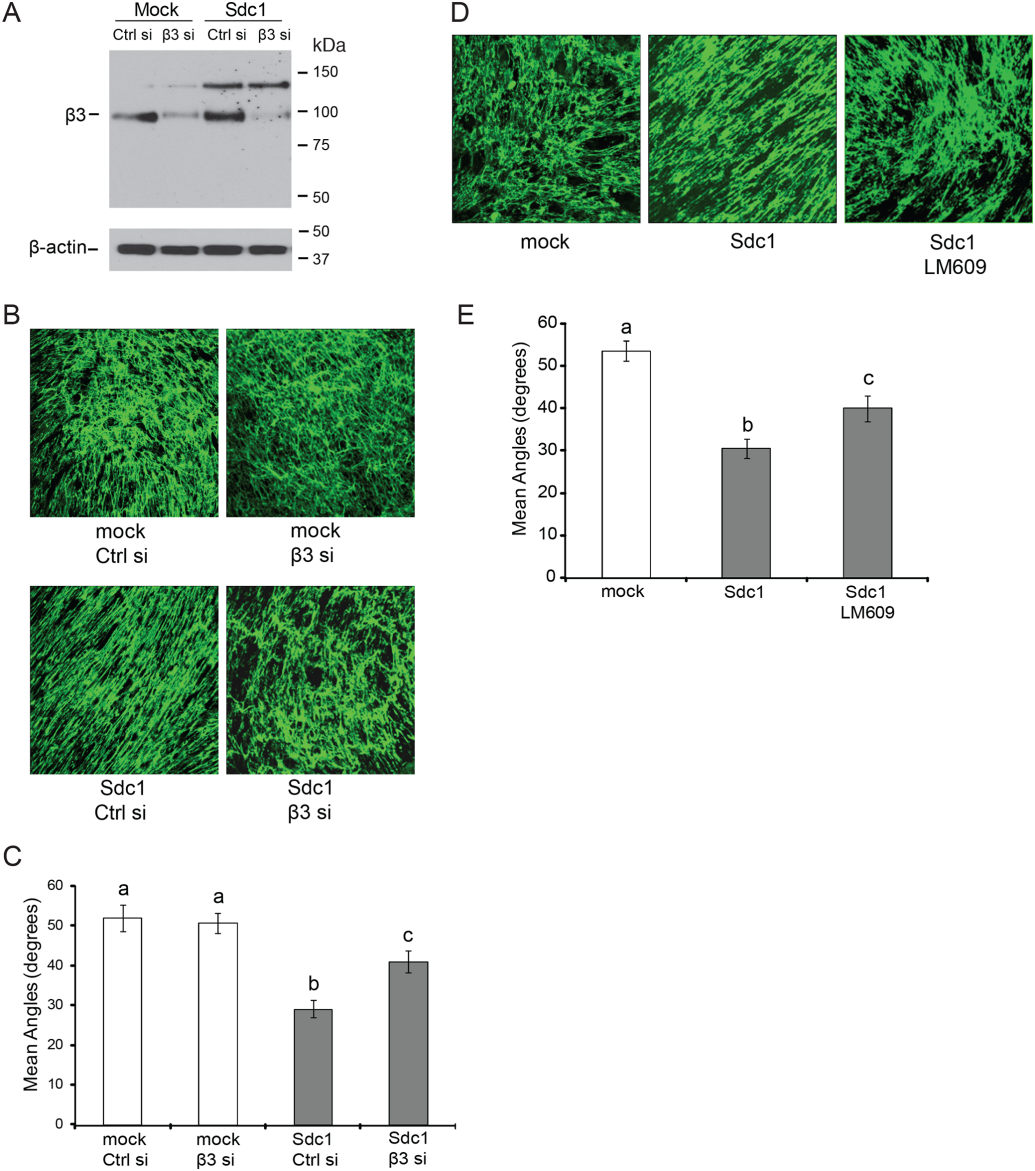
Integrin αvβ3 activity is required for Sdc1-mediated ECM fiber alignment. A-C) Silencing the expression of the integrin β3 subunit by siRNA diminishes ECM fiber alignment induced by Sdc1. A) Western blot analysis of integrin β3 subunit in HMF mock and HMF Sdc1 cells after treatment with control or β3 siRNA (125nM) for 3 days. B) Representative confocal microscopy images of ECMs labeled with an antibody to fibronectin (FN). The ECMs were derived from HMF-mock or HMF-Sdc1 cells treated with control or β3 siRNA. Original magnification: 200x. C) Mean fiber-to-fiber angles of ECMs produced by HMFs as indicated. Columns labeled with different letters are significantly different (at least p<0.01). D-E) Inhibition of integrin αvβ3 activity in HMF Sdc1 cells by function-blocking antibody LM609 significantly reduces FN fiber alignment. D) Representative confocal images of ECMs labeled with an antibody to FN. The ECMs were produced by HMF-mock, HMF-Sdc1 and HMF-Sdc1 treated with LM609 (30μg/ml). E) Mean fiber-to-fiber angles of HMF-derived ECMs as indicated. Columns labeled with different letters are significantly different (at least p<0.01). Ctrl si, control siRNA; β3 si, β3 siRNA.

As a first step towards this goal, we characterized the integrin subunit repertoire of HMF by Western blot analysis, which revealed expression of subunits α4, α5, αv, β1 and β3, whereas β4, β5 and β6 were undetectable (Fig 4A and S1 Fig). The presence of these subunits suggests that HMF carry integrins α4β1, α5β1, αvβ1 and αvβ3 on the cell surface. Among the integrins expressed by HMF, integrin αvβ3 is known to be activated by Sdc1 and to interact with the Sdc1 core protein via an integrin engagement site located within a 34–amino acid stretch of the Sdc1 ectodomain between residues 88 and 121 [31,32]–the core protein domain required for ECM fiber alignment in our model. Additionally, it has been reported that integrin αvβ3 has the ability to initiate and catalyze FN fibrillogenesis once properly activated [40,41]. Therefore, we explored the role of αvβ3 in Sdc1-mediated ECM alignment.

To suppress αvβ3 integrin activity, we silenced β3 subunit expression by siRNA. The β3 subunit forms heterodimers only with αv and αIIb subunits [42]. Since the expression of αIIb is restricted to platelets and megakaryocytes, expression silencing of the β3 subunit in HMF will only affect integrin αvβ3 activity. Western blot analysis showed that after treatment with siRNAs, the expression of the β3 subunit target was almost completely abolished in HMF mock and HMF Sdc1 cells (Fig 4B). Time course experiments revealed that a single transfection with β3 siRNAs (125nM) achieved efficient gene silencing that lasted as long as 7 days post-transfection (S2 Fig). Down-regulation of integrin αvβ3 activity in HMF Sdc1 cells diminished ECM fiber alignment in ECM-Sdc1 (Fig 4C). The mean fiber-to-fiber angles in ECMs produced by β3 siRNAs-treated HMF Sdc1 cells (ECM-Sdc1-β3 siRNAs) were significantly larger than those in ECM produced by control siRNA-treated HMF Sdc1 cells (ECM-Sdc1-control siRNA) (Fig 4D). In contrast, β3 knockdown in HMF mock had no effect on the topography of ECM produced by these cells. Similar results were obtained when αvβ3 activity was inhibited with a function-blocking antibody. Treatment of HMF Sdc1 cells with anti-αvβ3 antibody LM609 during the ECM production phase significantly reduced the FN fiber alignment (Fig 4E and 4F). The ECMs produced by HMF Sdc1 cells treated with either β3 siRNA or LM609 were somewhat more organized than ECM produced by HMF mock cells, which might be due to the functions of Sdc1 independent of αvβ3 activation or incomplete suppression of integrin αvβ3 activity by either approach. However, these results indicate that integrin αvβ3 activity is required for full Sdc1-induced ECM alignment.

**Fig 5:**
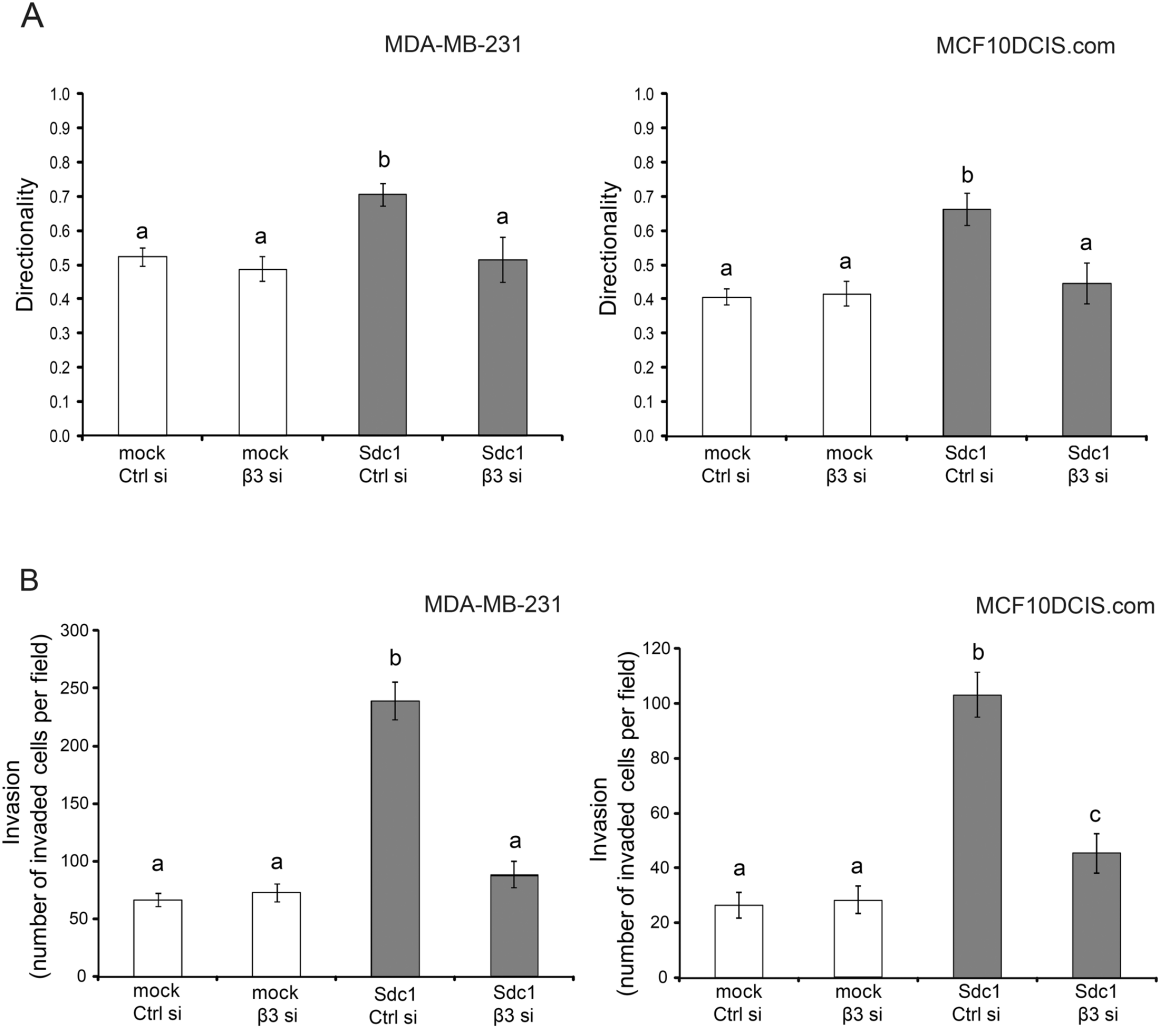
Silencing integrin β3 expression diminishes migration and invasion-modulating properties of the HMF-Sdc1-derived ECM. A) Quantitative assessment of migration directionality of MDA-MB-231 and MCF10DCIS.com cells within the ECMs produced by HMF-mock or HMF-Sdc1 cells treated with control or β3 siRNA using time-lapse microscopy. Different letters above columns indicate significant difference (p<0.001). B) Invasion of MDA-MB-231 and MCF10DCIS.com cells through ECMs produced by HMF-mock or HMF-Sdc1 cells treated with control or β3 siRNA. Different letters above columns indicate significant difference (at least p<0.05). Ctrl si, control siRNA; β3 si, β3 siRNA.

The functional properties of these ECMs with respect to breast carcinoma migration and invasion were consistent with the ECM fiber angle measurements. Breast carcinoma cells seeded into ECMs derived from HMF-Sdc1, which had been treated with β3 siRNA, migrated with limited directional persistence, which was indistinguishable from carcinoma cell migration in ECM-mock (Fig 5A). Consistent with these migration results, β3 siRNA treatment of HMF-Sdc1 resulted in the production of an ECM that had lost its permissiveness to the invasion of breast carcinoma cells (Fig 5B). The migration and invasion behavior of MCF10DCIS.com cells, a model of ductal carcinoma in situ (DCIS) [43], in these ECMs was very similar to MDA-MB-231 cells (Fig 5A and 5B), indicating that the functional ECM properties are relevant not only to one migratory cell line.

To test the specificity of αvβ3 integrin activity in determining ECM architecture and function, we examined the role of α4β1, another integrin present in HMF with FN binding activity and purported ability to participate in ECM fibrillogenesis [44]. Knockdown of the α4 subunit, and thus disruption of α4β1 activity, had no effect on Sdc1-dependent ECM fiber alignment, suggesting that αvβ3 indeed is specifically required to mediate the Sdc1 activity (S3 Fig).

### Integrin αvβ3 activity alone appears not to be sufficient for the generation of a fully migration and invasion-permissive ECM

**Fig 6:**
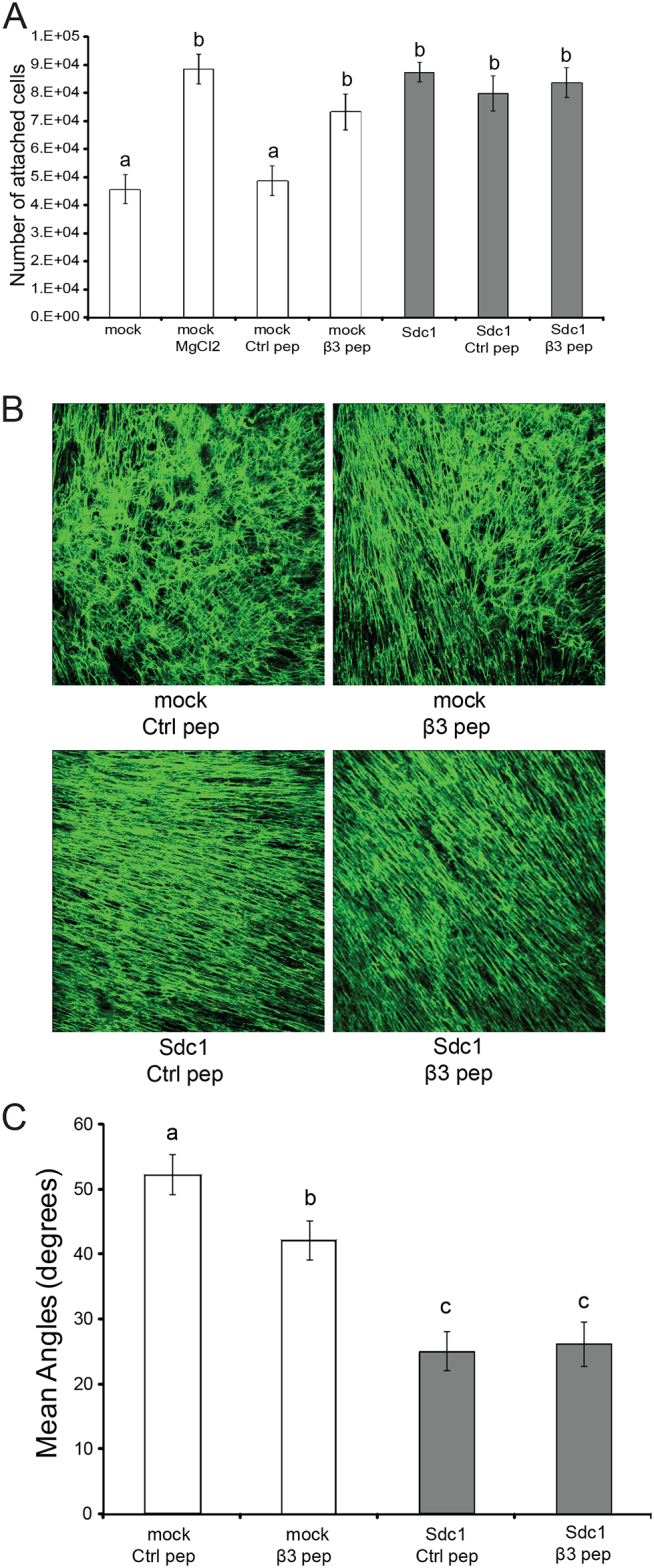
Forced activation of integrin αvβ3 leads to partial ECM fiber alignment. A) Activation of the αvβ3 integrin can be achieved independent of Sdc1 by β3 clasp peptide. Integrin αvβ3 activities were accessed by measuring the attachment of HMF cells, including untreated HMF cells, HMF cells treated with 250μM control peptide (ctrl pep), HMF cells treated with 250μM β3 clasp peptide (β3 pep) and HMF mock cells treated with 2 mM MgCl_2_, to culture dishes pre-coated with vitronectin after 15min incubation at 37°C. Columns labeled with different letters are significantly different (at least p<0.05). B) Representative confocal images of immunofluorescently labeled FN fibers of ECMs from HMF mock and Sdc1 cells treated with control or β3 clasp peptide. Original magnification: 200x. C) Mean fiber-to-fiber angles of the indicated HMF ECMs. Columns labeled with different letters are significantly different (at least p<0.05). Ctrl pep, control peptide; β3 pep, β3 clasp peptide.

To better understand the role of integrin αvβ3 in the generation of ECMs with tumor promoting properties, we tested whether the forced activation of αvβ3 would bypass the requirement for Sdc1. We chose to activate the integrin by treatment with β3 clasp peptide, an 8 amino acid peptide segment derived from the clasp region of the β3 subunit [45]. Integrins can switch between a low affinity and a high affinity conformation for ligand binding and it has been shown that the interaction between the clasp regions of the αv and β3 subunit locks the integrin αvβ3 in the low-affinity or inactive conformation. β3 clasp peptide disrupts this interaction and promotes the activation of the heterodimer [45]. To confirm the activating effect of the clasp peptide in HMF cells, a 15-minute attachment assay was performed. HMF Sdc1 cells attached more readily to surfaces coated with vitronectin (a αvβ3 substrate) than HMF mock cells (Fig 6A), indicating increased integrin αvβ3 activity in HMF Sdc1 cells. Exposure of HMF mock cells to the divalent cation Mg^2+^, a non-specific integrin activator, stimulated attachment to a level indistinguishable from HMF Sdc1. Treatment of HMF mock cells with β3 clasp peptide dramatically enhanced cell attachment to vitronectin, to a value that was comparable to HMF Sdc1 cells. In contrast, β3 clasp peptide treatment failed to further promote the attachment of the HMF Sdc1 cells, indicating that there was no additional activation of integrin αvβ3 in these cells. Scrambled control peptides had no effect on either cell type. We concluded from these attachment assay results that the β3 clasp peptide effectively activated integrin αvβ3 in HMF mock cells.

Activation of integrin αvβ3 in HMF mock cells by treatment with β3 clasp peptide (250μM) induced changes in the ECM architecture (Fig 6B and 6C). The mean fiber-to-fiber angle in ECMs produced by β3 clasp peptide-treated HMF mock cells was more acute than in ECMs produced by control peptide-treated HMF mock cells. However, compared to the ECMs produced by HMF Sdc1 cells treated with control peptide or β3 clasp peptide, the arrangement of the FN fibers was less aligned in ECMs derived from HMF-mock, treated with β3 clasp peptide.

**Fig 7:**
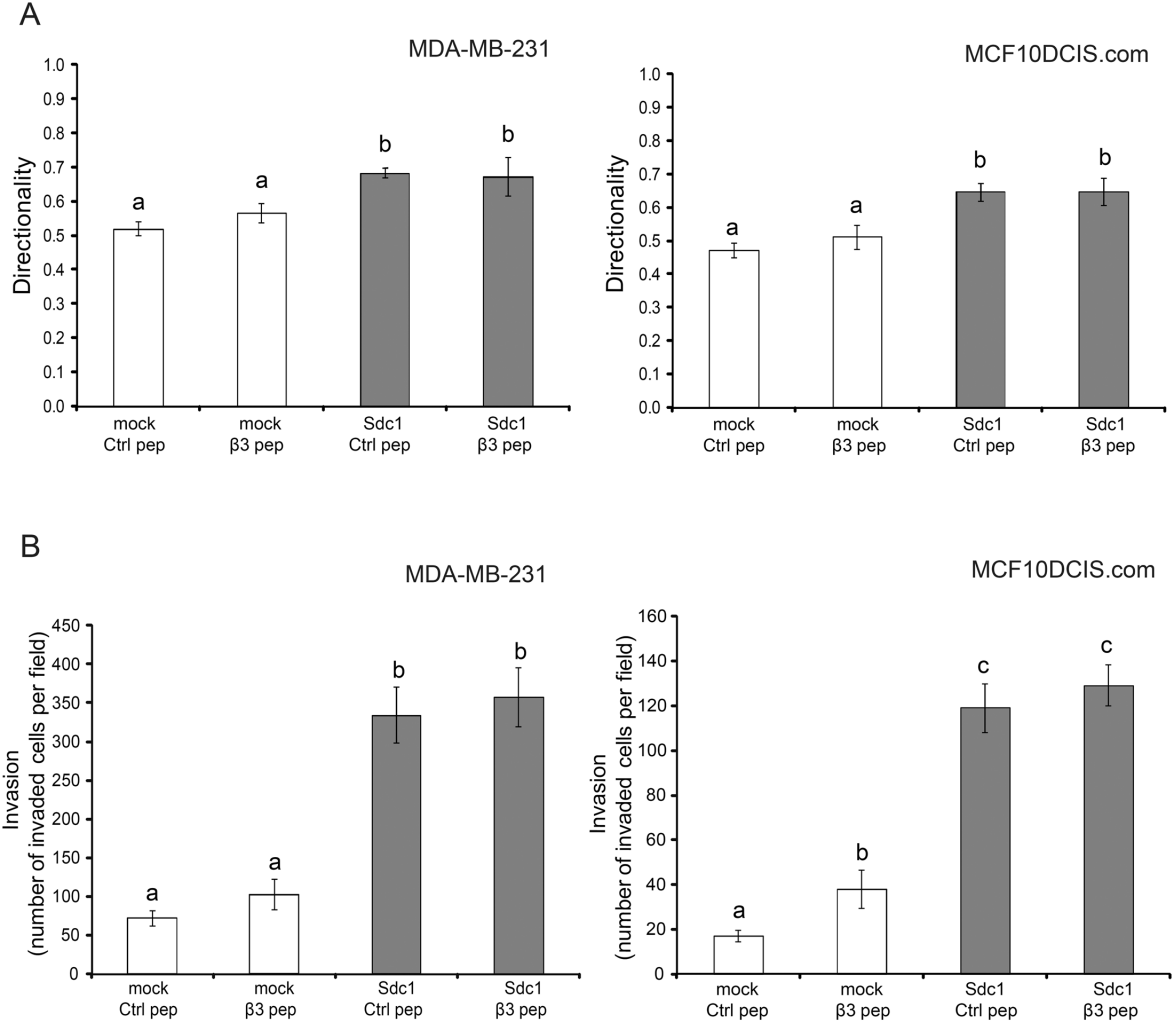
Activation of integrin αvβ3 alone is not sufficient for generating a migration and invasion-permissive ECM. A) Quantitative assessment of migration directionality of MDA-MB-231 and MCF10DCIS.com cells within the ECMs from HMF mock and Sdc1 cells treated with control or β3 clasp peptide using time-lapse microscopy. Columns labeled with different letters are significantly different (p<0.001). B) Invasion of MDA-MB-231 and MCF10DCIS.com cells through ECMs from HMF mock and Sdc1 cells treated with control or β3 clasp peptide. Columns labeled with different letters are significantly different (at least p<0.05). Ctrl pep, control peptide; β3 pep, β3 clasp peptide.

Invasion of MCF10DCIS.com cells through ECMs produced by β3 clasp peptide-treated HMF-mock was significantly increased compared to control-peptide-treated cells. However, β3 clasp peptide treatment of HMF failed to significantly enhance migration directionality of either MDA-MB-231 or MCF10DCIS.com cells or invasion of MDA-MB-231 cells (Fig 7A and 7B). Increasing the concentration of β3 clasp peptide to 500μM failed to further stimulate the invasion of breast carcinoma cells (N.Y., unpublished data). These results suggest that integrin αvβ3 activity is required but not sufficient for the generation of an ECM that permits directionally persistent migration and invasion of breast carcinoma cells. However, we can not entirely exclude the possibility that the clasp peptide induces suboptimal αvβ3 activation under the conditions permissive for ECM production.

### Integrin αvβ3 activity is involved in Sdc1-induced elongation of HMF cells when cultured in an overconfluent state during ECM production

**Fig 8:**
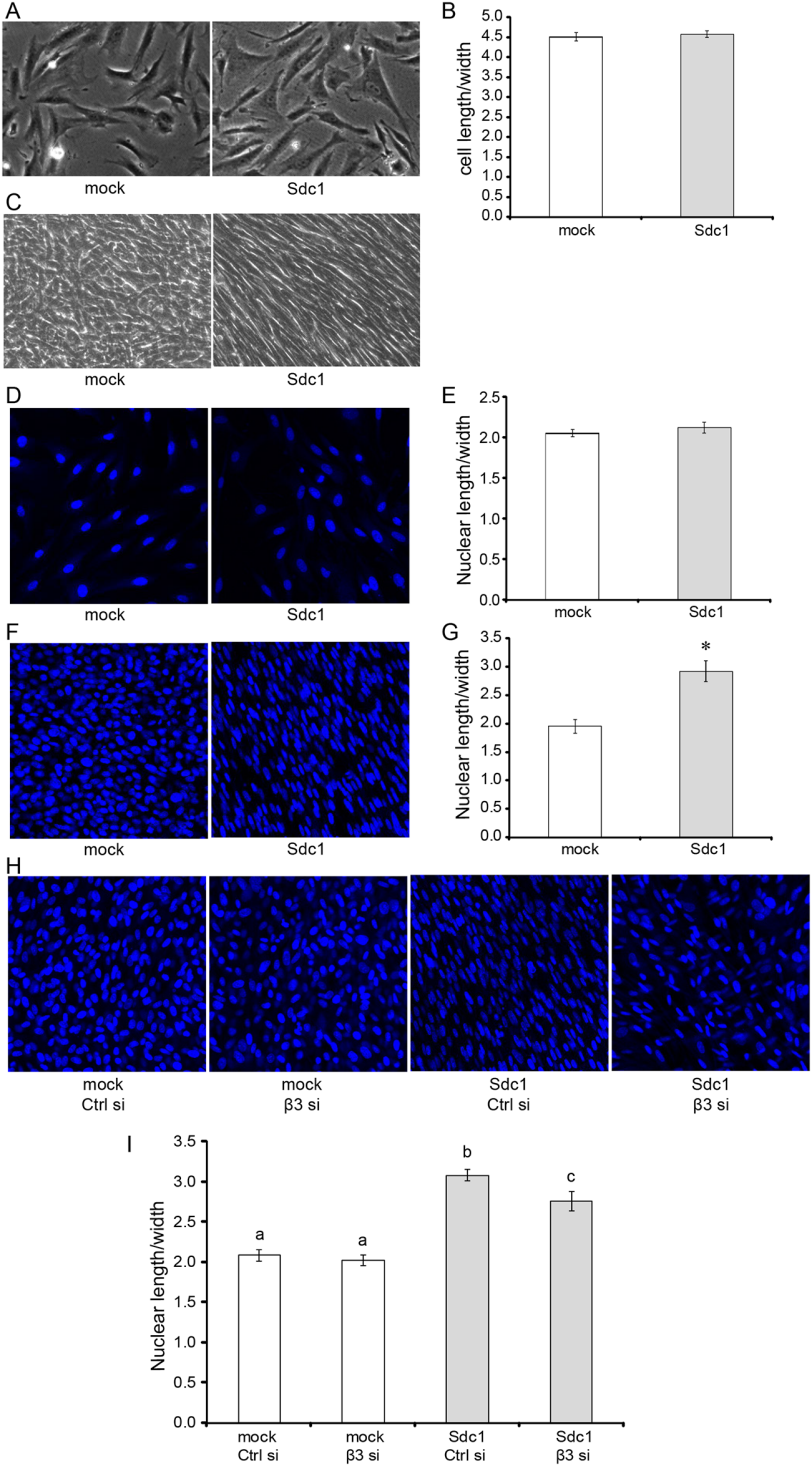
Integrin αvβ3 activity is involved in Sdc1-induced morphological changes during 3D ECM production. A-B, representative phase contrast images of HMF mock and Sdc1 cells cultured on gelatin-coated plates under semiconfluent states (A; original magnification: 100x) and the corresponding mean elongation indices (ratio of cell length/width) of HMF mock and Sdc1 cells (B). C, representative phase contrast images of HMF mock and Sdc1 cells after 7 d of culture under conditions permissive for 3D ECM production (Original magnification: 100x). D-E, representative confocal images of the nuclei of HMF mock and Sdc1 cells cultured on gelatin-coated plates under semiconfluent states (D; original magnification: 200x) and the corresponding mean nuclear elongation indices (ratio of nuclear length/width) of HMF mock and Sdc1 cells (E). The nuclei were labeled with Hoechst 33342. F-G, representative confocal images of the nuclei of HMF mock and Sdc1 cells after 7 d of culture under conditions permissive for 3D ECM production (F; Original magnification: 200x) and the corresponding mean nuclear elongation indices of HMF mock and Sdc1 cells (G). The nuclei were labeled with Hoechst 33342. *indicates that the difference was statistically significant (p<0.0001). H-I, representative confocal images of the nuclei of control siRNA or β3 siRNA treated HMF mock and Sdc1 cells after 7 d of culture under conditions permissive for 3D ECM production (H; original magnification: 200x) and the corresponding mean nuclear elongation indices (I). SiRNA treatment was performed as described in Material and Methods. The nuclei were labeled with Hoechst 33342. Letters above the columns indicate the results of statistical comparisons by ANOVA. Columns sharing the same letter are not significantly different; columns labeled with different letters are significantly different (at least p<0.05).

Cell morphology and orientation may affect ECM architecture. Syndecans play important roles in regulating cell morphology [29,46–48]. In addition, engagement of integrins may lead to actin cytoskeleton remodeling and alteration of cell morphology (for a review see [49]). Therefore, we examined and compared the morphology of HMF mock cells and HMF Sdc1 cells. HMF Sdc1 cells were morphologically indistinguishable from HMF mock when the cells were cultured on traditional two dimensional (2-D) substrate under a semiconfluent state (Fig 8A). The elongation indices of the HMF mock and HMF Sdc1 cells (ratio of cell length/width) were virtually identical (Fig 8B). However, clear morphological differences were detected between HMF mock and Sdc1 cells when the cells were cultured in an overconfluent state under the condition permissive for ECM production. Phase contrast images of ECM cultures of HMF cells showed that while HMF mock cells exhibited a more rounded cell shape, Sdc1-overexpressing HMF Sdc1 cells became much more elongated and gained a spindle-shaped morphology (Fig 8C). The morphological changes of HMF Sdc1 occurred early during ECM production. HMF Sdc1 became spindle-shaped after 2 days of culture under the conditions required to produce 3-D ECM (data not shown). We attempted to analyze the morphological changes of HMF Sdc1 cells quantitatively. However, HMF cells formed multiple layers during the 7 days of culture, making it impossible to clearly outline cell contours and therefore to determine elongation indices. It has been shown that cell shape is positively correlated with nuclear shape [50,51]. Therefore, we compared the nuclear shape and nuclear elongation indices (nuclear length/width) of HMF cells. Indeed, we found that there were no significant differences in nuclear shape and nuclear elongation indices between HMF mock and Sdc1 cells when the cells were cultured on 2-D substrate in a semiconfluent state (Fig 8D and 8E). However, the nuclei of HMF Sdc1 cells became greatly elongated after 7 days of culture under conditions permissive for ECM production (Fig 8F). The nuclear elongation index of HMF Sdc1 cells was markedly increased and became significantly larger compared to the nuclear elongation index of HMF mock cells (Fig 8G). Moreover, knockdown of the expression of integrin β3 subunit by siRNA in Sdc1-overexrpessing HMF Sdc1 cells resulted in a less elongated nuclear morphology when the cells were cultured in an overconfluent state under the conditions permissive for ECM production (Fig 8H). The nuclear elongation index of HMF SDC1 cells treated with siRNA against integrin β3 subunit was significantly decreased in comparison to HMF Sdc1 cells treated with control siRNA (Fig 8I). The results suggest that Sdc1 overexpression induces morphological changes in HMF cells through αvβ3 integrin, which may account for one of the mechanisms by which Sdc1 mediates ECM fiber alignment.

## Discussion

A growing body of evidence indicates that composition and physical properties of the ECM are altered in breast cancer and that these changes contribute to cancer progression [7,8,13,52]. Much of this work has focused on ECM density and rigidity [7,13,53] but more recently, ECM fiber alignment also has been shown to predict invasion and metastasis [11,25,54]. The Cukierman group reported that murine skin tumor fibroblasts produce an ECM with aligned, parallel fibers when cultured in 3D [55]. Work by Provenzano and colleagues demonstrated that in Wnt-1 and PyMT-induced mouse mammary tumors, collagen fibers organize progressively around the tumor islets [11]. As the tumor becomes invasive, collagen fibers become aligned and eventually orientated perpendicularly to the advancing edge of the tumor. This collagen fiber organization, termed by the investigators “tumor-associated collagen signature-3” (TACS-3), facilitates the invasion of tumor cells into the stroma and metastasis. Moreover, the presence of TACS-3 features serves as a prognostic indicator of poor outcome in human breast cancer [54]. The Tlsty group found that transformed mammary epithelial cells or carcinoma cells assume a mesenchymal phenotype when co-cultured and metastasize more readily when co-inoculated with carcinoma-associated fibroblasts (CAF) compared to reduction mammoplasty fibroblasts (RMF), a difference that could be attributed to the deposition of a distinct, aligned ECM [56]. The ECM architecture may also mediate the effects of pregnancy and lactation on mammary tumor development. For example, the spatial organization of fibrillar collagen, which is haphazardly arranged in glands of parous rats and more linearized/aligned in glands of nulliparous animals, is the main determinant of whether mammary ECM suppresses or promotes tumor cell invasion [57].

To date, the mechanism by which the architecture of the ECM fibers is regulated has remained largely unknown. Recently, we have identified Sdc1 as a potent regulator of ECM architecture [25]. While Sdc1 expression in stromal fibroblasts is not required for ECM production, its presence alters ECM architecture *in vivo* and *in vitro*, resulting in an aligned, parallel ECM fiber organization that promotes the directional migration and invasion of breast carcinoma cells. Other syndecan family members have also been implicated in ECM assembly. Sdc2 promotes the production of a FN matrix in CHO cells by activating various integrins [58]. Sdc4 participates in regulating FN fibrillogenesis by associating with α5β1 integrin and possibly by activating Rho and protein kinase C (PKC) [59]. In corneal stromal cells of the eye, Sdc1 is required for efficient FN fibrillogenesis but becomes dispensable when integrins are generically activated by MnCl_2_, suggesting that the role of Sdc1 in ECM assembly involves the regulation of integrin activity [60]. Curiously, in the same study, Sdc1 deficiency resulted in increased FN fibrillogenesis in fibroblasts.

In the present study, we explored the mechanisms by which Sdc1 modulates ECM architecture. Our data show that HMF cells overexpressing Sdc1 exhibit a more elongated, spindle-shaped cell morphology when the cells were cultured in an overconfluent state under the conditions permissive for ECM production, which is significantly different from the morphology of mock transfected HMF cells. Similar morphological differences have been observed between cultures of tumor-associated fibroblasts and normal fibroblasts, which produce aligned ECM and randomly organized ECM respectively [55]. In addition, FAP+ fibroblasts that organize ECM fibers in parallel patterns are also found to become elongated into an enhanced spindled shape [61]. Goetz et al observed that caveolin-1, a caveolar scaffolding protein, is required for fibroblast elongation, efficient ECM fiber alignment and the elongation of cells seeded into these matrices [62].

Cell morphology and orientation affects ECM architecture. We have shown previously that mammary fibroblasts, which are forced to elongate and align by plating them on patterned FN surfaces, produce an ECM with an aligned fiber architecture [25]. Therefore, it is likely that the altered cell morphology induced by Sdc1 plays a role in the production of an aligned ECM. Sdc1 directly interacts with and activates the integrin αvβ3 via its ectodomain [32,63]. It is well known that engagement of integrins may lead to the remodeling of the actin cytoskeleton and an alteration of cell shape (for a review see [64]). Therefore, Sdc1 may modulate cell morphology though activating integrin αvβ3, thereby altering ECM architecture. Our findings that downregulation of the expression of integrin β3 subunit by siRNA in Sdc1-overexpressing HMF cells leads to a less elongated cell morphology and more random ECM organization are consistent with the above hypothesis. However, we cannot exclude the possibility that the morphological change of HMF Sdc1 cells is the result rather than the cause of ECM alignment. HMF cells continue to produce ECM during the 7-day culture period. ECM topography and composition influence cell morphology. It has been demonstrated that the aligned ECM produced by tumor associated fibroblasts is sufficient to induce a spindle-shaped morphology in normal primary fibroblasts [55].

It is also possible that Sdc1 induces ECM fiber alignment by modulating FN fibrillogenesis. FN is the major component of the provisional ECM into which other ECM components such as collagens, fibulin-1, thrombospondin-1 and tenascin-C are then integrated (reviewed in [65]). By regulating the topography of the FN-rich provisional ECM, Sdc1 may govern the architecture and function of the mature fibroblast-derived ECM. FN fibrillogenesis is a complex, cell-driven process in which cell surface receptors such as integrins and HSPGs play essential roles. FN is composed of three types of repeating modules, which include 12 type I repeats, 2 type II repeats, and 15 type III repeats. FN is secreted as compactly folded, disulfide-bonded dimers that are functionally inactive. Binding of FN dimers to integrins at the cell surface triggers a series of cellular events, eventually resulting in conformational changes in FN that unravel the cryptic FN self-association sites and convert the molecule into an extended and active form (for reviews, see [65–67]). The requirement of Sdc1 HS chains and integrin αvβ3 in our experiments suggests that stromal Sdc1 may modulate FN fibrillogenesis by both direct and indirect mechanisms. Two high affinity heparin binding domains, Hep I and Hep II, have been identified in FN, which interact with the HS chains of various members of the syndecan family [68,69]. Hep I and Hep II domains map to the type I_1–5_ and type III_12–14_ repeats, respectively [70]. It has been shown that the type I_1–5_ repeats also harbor a major site for FN self-assembly and the type III_12–14_ repeats are essential for the intramolecular interactions that maintain the compact, globular conformation of FN dimers [71,72]. Once expressed by stromal fibroblasts, Sdc1 may in part directly regulate the unfolding of dimeric FNs and the interactions between cell-associated FN dimers through its HS chains, thus affecting the architecture of the multimeric FN fibrils.

Sdc1 directly interacts with and activates the integrin αvβ3 via its ectodomain [32,63]. While the assembly of FN fibril matrix appears to be initiated mainly by integrin α5β1 [37,73], integrin αvβ3 also has the ability to support this process once properly activated [40,41,74]. Integrin α5β1 and αvβ3 differ in their requirements for binding sites on FN. Integrin α5β1 requires a synergy region located in type III_9_ repeats in addition to the Arg-Gly-Asp (RGD) motif for obtaining maximal binding to FN, whereas αvβ3 utilizes a binding site located in N-terminal type I_1–5_ repeats besides the RGD motif [75–78]. Moreover, there is evidence that FN fibrils generated by αvβ3 integrin activity display distinct features from those produced by α5β1 [40,74]. It is possible that in Sdc1-negative fibroblasts, α5β1 functions as the major integrin for the initiation of FN fibrillogenesis, forming an intricate meshwork of FN fibrils. In fibroblasts overexpressing Sdc1, the activity of integrin αvβ3 is greatly increased. The initiation of FN fibrillogenesis in Sdc1-overexpressing fibroblasts may be conducted mainly by integrin αvβ3, which, in combination with the effects of HS chains of Sdc1, results in the production of an aligned ECM that is different from the ECM assembled under the control of α5β1. In this context, it is interesting that the stromal ECM of carcinomas growing in β3 integrin deficient mice contains thicker, laterally fused collagen fibers, contrasting with the delicate fibers seen in wild-type animals [79]. How these findings relate to the Sdc1-dependent fiber alignment is uncertain but they are congruent with our data by demonstrating a dramatic effect of β3 integrin activity on ECM fiber architecture *in vivo*.

**Fig 9:**
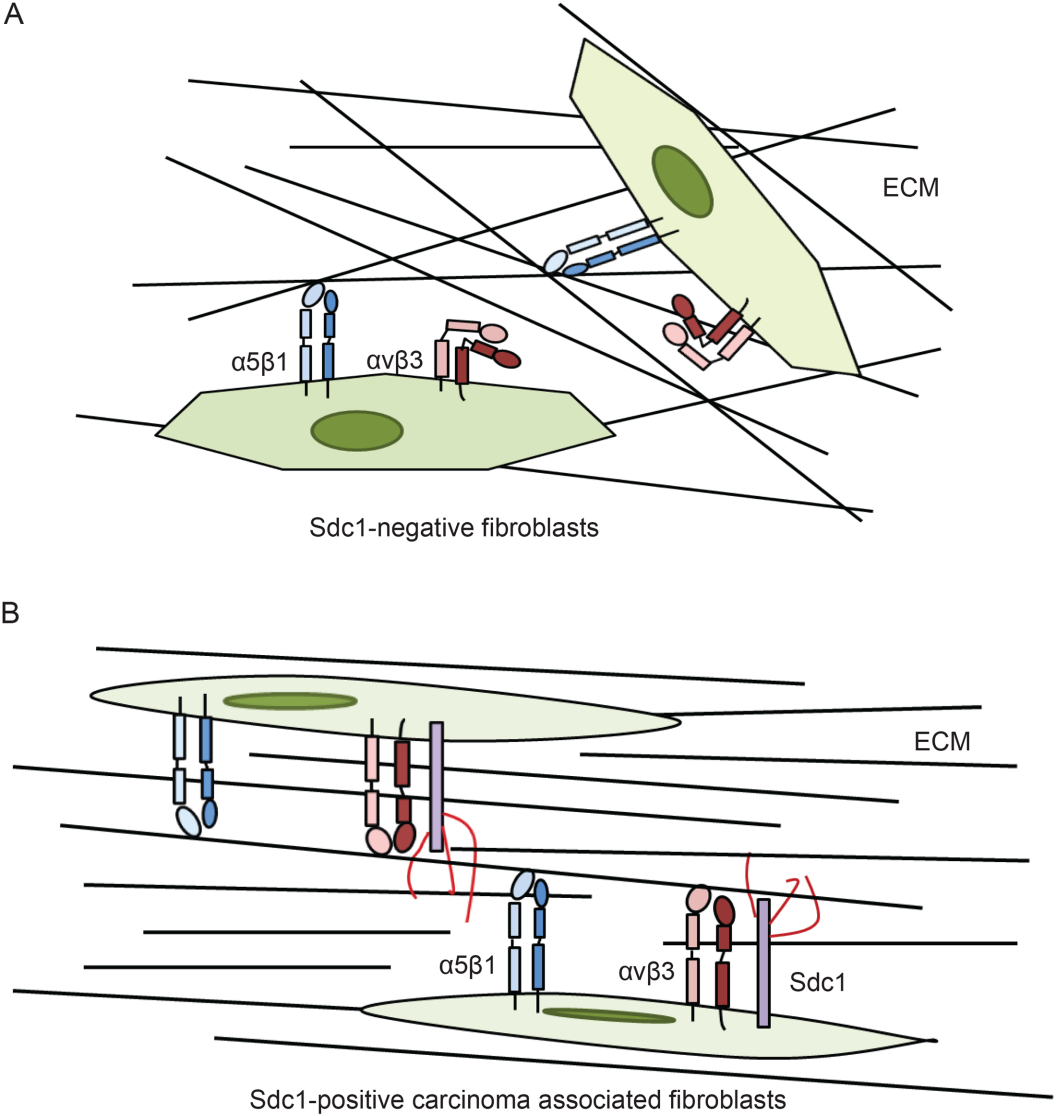
Potential molecular mechanisms by which stromal Sdc1 regulates ECM fiber alignment. A) In Sdc1-negative normal fibroblasts, FN fibrillogenesis is likely initiated by integrin α5β1, which gives rise to an ECM with haphazard fiber arrangement. B) In the stromal fibroblasts of some invasive breast carcinomas, Sdc1 is aberrantly expressed. Sdc1 induces morphological changes of the fibroblasts, which may contribute to the production of aligned ECM. Sdc1 may also modulate FN fibrillogenesis by directly regulating the unfolding of dimeric FNs and the interactions between cell-associated FN dimers through its HS chains. In addition, Sdc1 may modulate the initiation of FN fibrillogenesis by activating integrin αvβ3 through its ectodomain. The combined effects of Sdc1 affect the architecture of the multimeric FN fibrils, resulting in an ECM with an organized, aligned fiber arrangement.

In summary, we have demonstrated a regulatory effect of Sdc1 on the tumor ECM architecture. Sdc1 is aberrantly expressed in stromal fibroblasts of invasive breast carcinomas, leading to an aligned ECM fiber architecture. Our data suggest that multiple mechanisms may contribute to Sdc1-induced ECM alignment. Sdc1 expression alters the morphology of fibroblasts through integrin αvβ3. In addition, Sdc1 may modulate the assembly of 3D fibrillar ECM by directly interacting with FN via its HS chains and by activating integrin αvβ3 via its ectodomain (shown schematically in Fig 9). These ECM remodeling events convert the stroma from an invasion-restrictive to an invasion-permissive microenvironment and create “highways” that may facilitate the invasion and spreading of breast carcinoma cells.

## Materials and Methods

### Cell culture

Immortalized human mammary fibroblasts were kindly provided by Dr. C. Kuperwasser [80]. The human breast carcinoma cell lines MDA-MB-231 and MCF10DCIS.com [43] were purchased from ATCC (Manassas, VA) and Asterand (Detroit, MI), respectively. Stable cell lines derived from HMF that expressed wildtype and mutant forms of Sdc1 or empty plasmid were maintained in DMEM supplemented with 10% calf serum, 2 mM L-glutamine, 100U/mL penicillin/streptomycin and 250 μg/ml G418. MDA-MB-231 cells were cultured in DMEM supplemented with 10% fetal bovine serum, 2 mM L-glutamine and 100U/mL penicillin/streptomycin. MCF10DCIS.com cells were maintained in DMEM/F12 medium containing 5% horse serum and 100U/mL penicillin/streptomycin.

### Stable transfections

The generation of mock-transfected HMF and HMF stably transfected with Sdc1 has been described previously [25]. Mammalian expression vectors (pcDNA3.1) containing the cDNAs encoding truncated and mutated mouse Sdc1 were generous gifts from Dr. Ralph Sanderson (University of Alabama, Birmingham) and Dr. Alan Rapraeger (University of Wisconsin-Madison, Madison, WI). The Sdc1 mutant constructs included a heparan sulfate-deficient mutant (Sdc1-ΔHS) [28], an ectodomain truncation mutant (Sdc1-ΔEcto) [32], a transmembrane domain substitution mutant (Sdc1-TMM) [29] and a cytoplasmic domain deletion mutant (Sdc1-ΔCyto) [30]. The syndecan-1 mutant constructs were stably transfected into HMF cells using the Amaxa System (Lonza, USA) according to the manufacturer’s instructions. Following transfection, cells were selected with 500 g/ml G418 and cells that expressed mutant Sdc1 at levels similar to wild-type Sdc1 were enriched by fluorescence-activated cell sorting (FACS).

### 3D ECM production

Preparation of the HMF-derived, *in vivo*-like ECM was performed according to the protocol developed by Cukierman and has been described previously [25,55,81]. Briefly, HMF cells were cultured in a highly confluent state for 7 days in the presence of 50 μg/ml sodium ascorbate (Fisher Scientific, Inc., Pittsburgh, PA) and then removed by alkaline detergent treatment (25 mM Tris-HCl, pH 7.4, 150 mM NaCl, 0.5% Triton X-100, and 20 mM NH_4_OH), leaving a cell-free 3D ECM attached to the culture surface.

### Analysis of ECM fiber orientation/alignment

The architecture of HMF-derived ECMs was visualized by immunofluorescence staining of the fibronectin (FN) fibers. The cell-free 3D ECMs were blocked with 5% FBS for 1 h at room temperature and then incubated with mouse anti-FN (BD Biosciences, San Juan, CA) antibody at 4°C overnight. After incubation with Alexa Fluor488 conjugated goat anti-mouse IgG for 1 h at room temperature, the ECM preparations were imaged with a laser-scanning confocal microscope. As previously described, the fiber-to-fiber angles of the ECMs were determined manually by overlaying the ECM images with a pre-designed template that defined nine evenly distributed measurement points. The fiber closest to each of the measurement points was identified and the intersection angle of the nearest crossing fiber was measured using the angle measurement tool of ImageJ (http://rsbweb.nih.gov/ij/) [25]. At least 90 fiber-to-fiber angles were measure for each condition/group.

### siRNA transfections

Knockdown of integrins was achieved by using the ON-TARGET*plus* SMARTpool siRNAs (Thermo Scientific, Rockford, IL). The ON-TARGETplus Non-targeting Pool served as negative controls. Transfection of the siRNAs was performed using Dharmafect 3 transfection reagent according to the manufacturer’s instructions (Thermo Scientific). Briefly, semiconfluent HMF cells were incubated with the transfection mixture containing 100-125nM of siRNAs for 72 hr. Cells were then harvested for Western blot analysis to confirm the knockdown of integrin expression and for the production of the 3D ECMs.

### Western blot analysis

Whole cell lysates of HMF cells were prepared using RIPA buffer (Boston BioProducts). Equal amounts of the resulting protein lysates were fractionated on 4–12% Criterion^™^ XT precast gel (Bio-Rad Laboratories, Inc) prior to transfer to polyvinylidene difluoride (PVDF) membranes. The membranes were probed with rabbit anti-human integrin α4 or rabbit anti-human integrin β3 antibodies (Cell Signaling Technology, Inc) at 4°C overnight, washed in 1xPBS, and then incubated with horseradish peroxidase-conjugated anti-rabbit IgGs (Sigma) at room temperature for 1 hr. The integrin α4 or integrin β3 subunit was visualized using the SuperSignal West Femto maximum sensitivity substrate (Pierce).

### Integrin stimulation

β3 clasp peptide and its scrambled control were designed based on published reports by Vomund *et al*. [45] and synthesized and purified by Biomatik (Wilmington, DE). The activation of integrin αvβ3 by β3 clasp peptide was confirmed using a 15-minute cell attachment assay. HMF cells were harvested and incubated with control or β3 clasp peptide for 30 minutes. Cells were then added to culture dishes precoated with the integrin αvβ3 substrate vitronectin and incubated at 37°C for 15 minutes. The unattached cells were removed by washing with PBS and attached cells were collected and counted using a hemocytometer. To investigate the effect of integrin αvβ3 activation on the architecture of the ECM, control and β3 clasp peptide were added to the medium throughout the process of matrix production.

### Cell migration analysis (time-lapse motility assay)

Live MDA-MB-231 and MCF10DCIS.com cells were fluorescently labeled using the CellBrite cytoplasmic membrane staining kit (Biotium, Inc., Hayward, CA) according to the manufacturer’s instructions. Labeled cells were seeded into glass-bottom tissue culture plates (MatTek Corporation, Ashland, MA) precoated with cell-free ECMs derived from HMF cells and incubated overnight. Cell movements were recorded every 30 minutes for a period of 5–6 hours on the BD Pathway confocal bioimager (BD Biosciences). The resulting images were stacked using the ImageJ software and the movement of individual cells was monitored by tracing the path of the manually detected cell center using the Fragment Line tool of ImageJ. The directional persistence of migration was determined as the ratio of net distance between starting point and end point to the total distance traveled.

### Invasion assay

HMF cells were cultured in the insert of BD Biocoat Matrigel Invasion Chambers (BD Biosciences) under conditions conducive to ECM production for 7 days. The cells were then removed to leave cell-free ECMs on the upper side of the insert. Breast carcinoma cells MDA-MB-231 or MCF10DCIS.com were seeded into these ECMs and cultured in DMEM supplemented with 2% calf serum or DMEM/F12 containing 1% horse serum for 24 hours. The lower chambers were filled with DMEM containing 10% calf serum or DMEM/F12 containing 5% horse serum as a source of chemoattractants. Non-invading carcinoma cells remaining on the upper side of the insert were removed. The invading cells attached to the lower side of the insert were fixed with 100% methanol and stained with Hoechst 33342 DNA dye. Images of the stained nuclei were acquired using an Olympus inverted microscope and analyzed using ImageJ software.

### Nuclear staining

HMF cells cultured in glass-bottom dishes were fixed with ice-cold 100% methanol for 15 min. Subsequently, the cells were incubated with the cell-permeable nucleic acid stain Hoechst 33342 (20μg/ml) for 30 min. The stained nuclei of HMF cells were imaged using the Nikon A1R-Si laser scanning confocal spectral microscope.

### Quantitative analysis of the morphology of HMF cells

Cellular and nuclear morphology of HMF cells were evaluated by calculating the elongation indices (length-to-width ratios) of the cells and their nuclei. HMF cells were either cultured on gelatin-coated glass-bottom plates at a semi-confluent state overnight or cultured in a highly confluent state for up to 7 days under conditions required for 3D ECM production. Phase contrast images of the HMF cells and confocal images of the Hoechst-stained HMF cells were analyzed using ImageJ software. Cell length was defined as the longest distance between any two points on the cell contour. Cell width was defined as the longest distance within the cell body perpendicular to the length. Nuclear length and width were defined as the length of the major (long) axis and minor (short) axis of the nucleus, respectively.

### Statistics

All the experiments were repeated at least two times. The experimental results were presented as mean ± standard deviation and statistically analyzed using the InStat 3.1 software (GraphPad Software, Inc., USA). Multiple paired comparisons were conducted using one-way ANOVA followed by Tukey's post-hoc test. P values of less than 0.05 were considered statistically significant unless otherwise indicated.

## Supporting Information

S1 Fig Western blot analysis of various integrin subunits in HMF mock and HMF Sdc1 cells. HMF cells express integrin subunits α4, α5, αv, β1 and β3.
(TIF)

S2 Fig Western blot analysis of integrin β3 subunit in HMF mock and HMF Sdc1 cells. Treatment with β3 siRNA (125 nM) successfully suppresses the expression of β3 subunit in HMF cells even 7 days after siRNA transfection.
(TIF)

S3 Fig Silencing the expression of integrin α4 subunit has no effect on Sdc1-induced ECM fiber alignment. A, Western blot analysis of the level of integrin α4 subunit in HMF mock and HMF Sdc1 cells after treated with 100nM control or α4 siRNA for 3 days. B, representative confocal images of immunofluorescently labeled FN fibers of ECMs from HMF mock and Sdc1 cells treated with control or α4 siRNA. Original magnification: 200x. C, mean fiber-to-fiber angles of indicated HMF ECMs. Columns labeled with different letters are significantly different (p<0.001). Ctrl si, control siRNA; α4 si, α4 siRNA.
(TIF)
