# Supplementary figures and images for: Syndecan-1-Induced ECM Fiber Alignment Requires Integrin αvβ3 and Syndecan-1 Ectodomain and Heparan Sulfate Chains
Source: PLoS One. 2016 Feb 24;11(2):e0150132. doi: 10.1371/journal.pone.0150132 (PMC4766302; doi:10.1371/journal.pone.0150132)

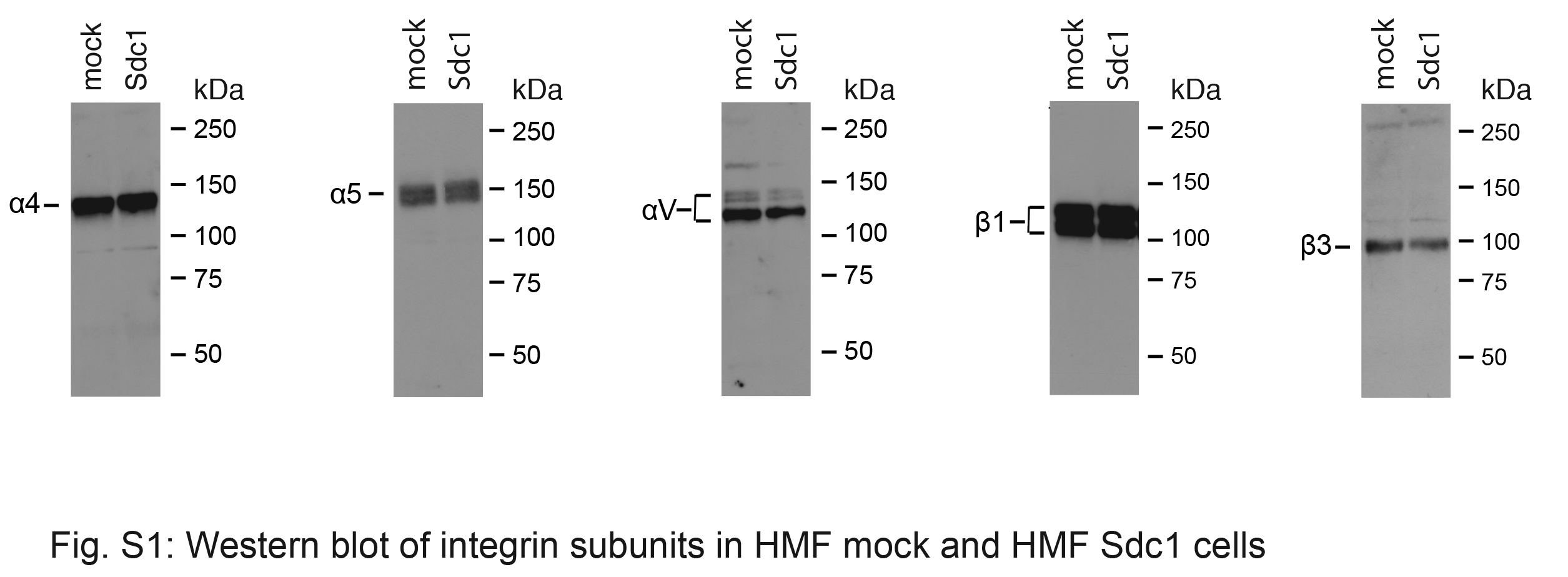

Supplement: S1 Fig — HMF cells express integrin subunits α4, α5, αv, β1 and β3. (TIF) [file pone.0150132.s001.tif]

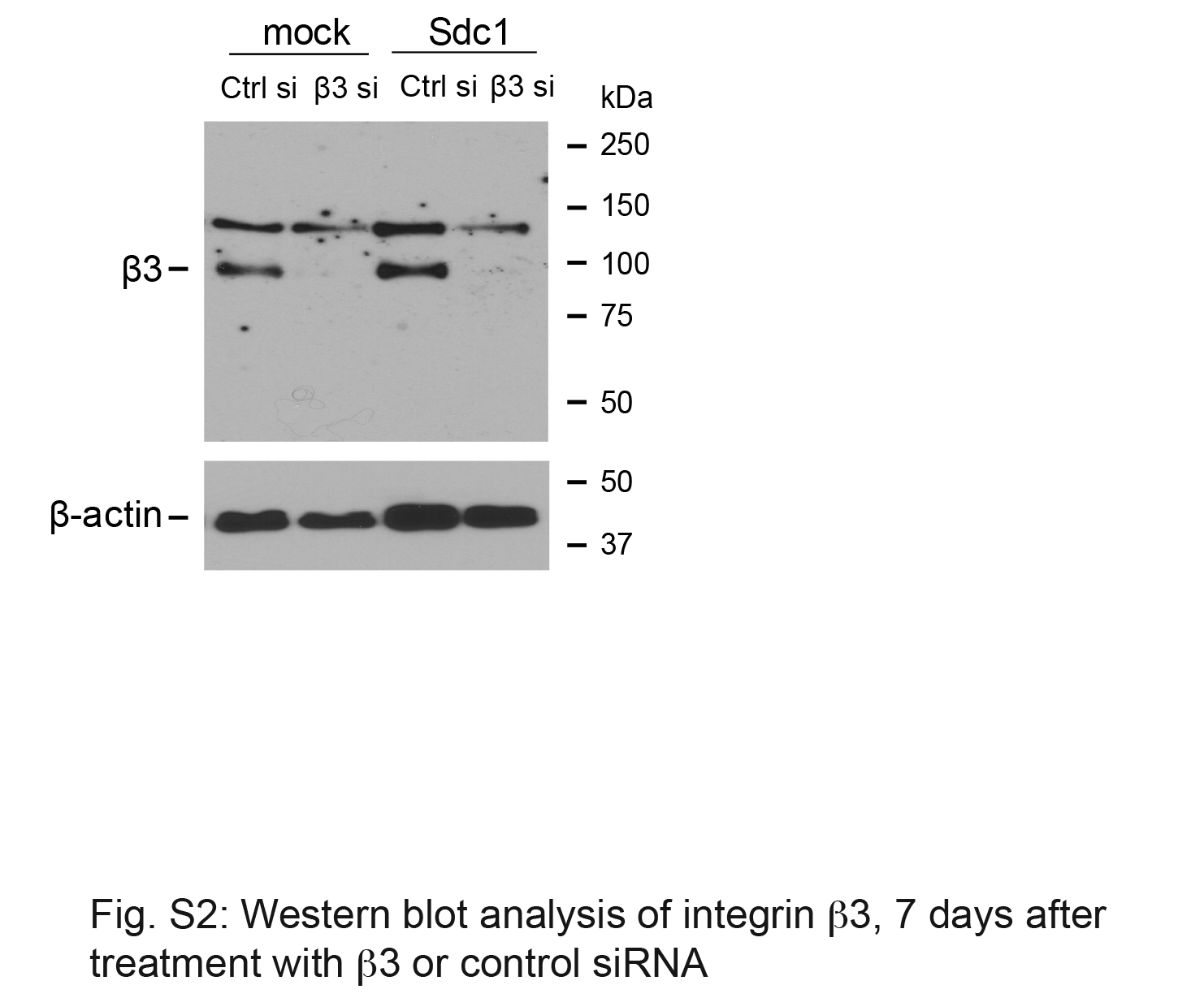

Supplement: S2 Fig — Treatment with β3 siRNA (125 nM) successfully suppresses the expression of β3 subunit in HMF cells even 7 days after siRNA transfection. (TIF) [file pone.0150132.s002.tif]

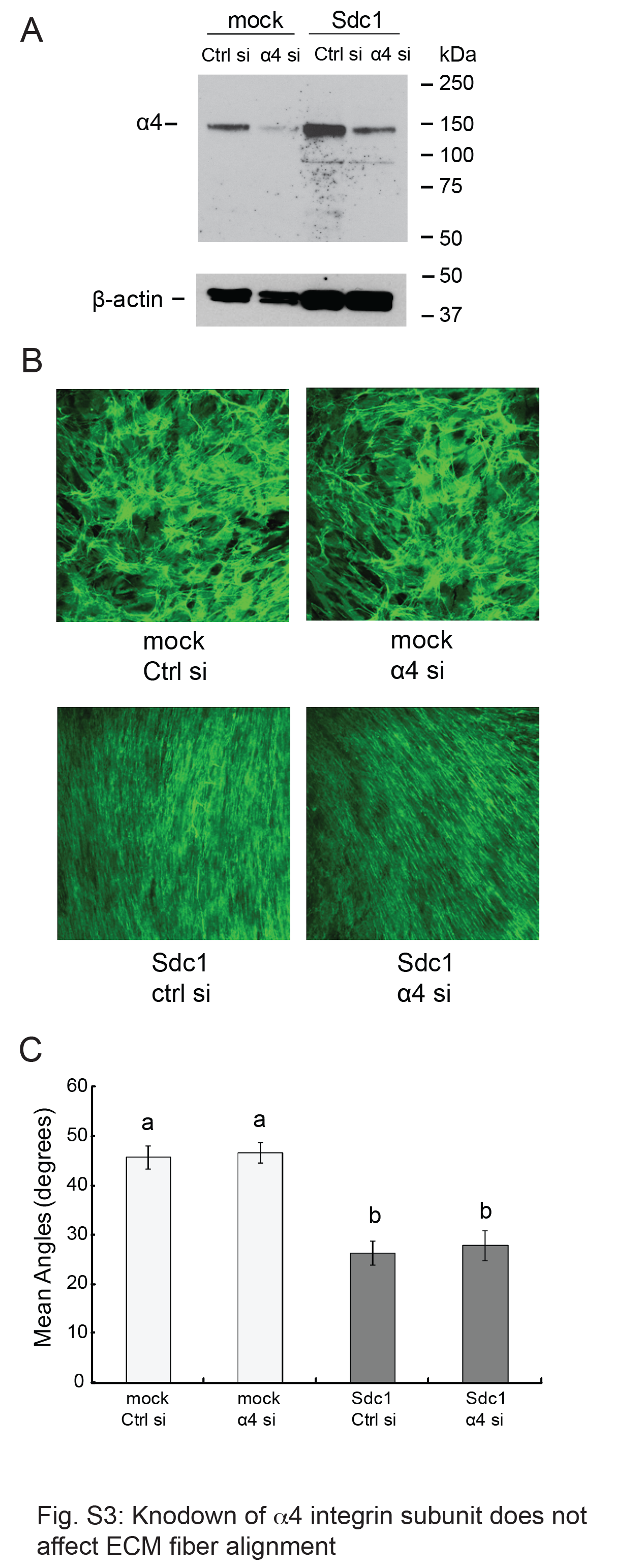

Supplement: S3 Fig — A, Western blot analysis of the level of integrin α4 subunit in HMF mock and HMF Sdc1 cells after treated with 100nM control or α4 siRNA for 3 days. B, representative confocal images of immunofluorescently labeled FN fibers of ECMs from HMF mock and Sdc1 cells treated with control or α4 siRNA. Original magnification: 200x. C, mean fiber-to-fiber angles of indicated HMF ECMs. Columns labeled with different letters are significantly different (p<0.001). Ctrl si, control siRNA; α4 si, α4 siRNA. (TIF) [file pone.0150132.s003.tif]
